# Detection and Quantification of the Harmful Dinoflagellate *Margalefidinium polykrikoides* (East Asian Ribotype) in the Coastal Waters of China

**DOI:** 10.3390/toxins14020095

**Published:** 2022-01-25

**Authors:** Jiarong Hu, Ruoyu Guo, Douding Lu, Xinfeng Dai, Yuanli Zhu, Bum Soo Park, Pengbin Wang

**Affiliations:** 1 Key Laboratory of Marine Ecosystem Dynamics, Second Institute of Oceanography, Ministry of Natural Resources, Hangzhou 310012, China; jiaronghu1997@163.com (J.H.); dinoflagellate@sio.org.cn (R.G.); doudinglu@sio.org.cn (D.L.); xinfengdai@sio.org.cn (X.D.); zyl0218@163.com (Y.Z.); 2 Key Laboratory of Tropical Marine Ecosystem and Bioresource, Fourth Institute of Oceanography, Ministry of Natural Resources, Beihai 536000, China; 3 Guangxi Key Laboratory of Beibu Gulf Marine Resources, Environment and Sustainable Development, Fourth Institute of Oceanography, Ministry of Natural Resources, Beihai 536000, China; 4 Marine Ecosystem Research Center, Korea Institute of Ocean Science & Technology, Busan 49111, Korea; parkbs@kiost.ac.kr

**Keywords:** *Margalefidinium polykrikoides* (East Asian ribotype), quantitative real-time PCR, field application, China coastal waters

## Abstract

As a marine ichthyotoxic dinoflagellate, *Margalefidinium polykrikoides*, previously named *Cochlodinium polykrikoides*, have caused mass mortalities of fish worldwide during blooms. Rapid detection of target species is a prerequisite for the timely monitoring and early warning of harmful algal blooms (HABs). However, it is difficult to achieve rapid identification with traditional methods. The technology of using quantitative real-time PCR (qPCR) to detect and quantify microalgae is relatively mature. Based on the accuracy, rapidity, and sensitivity of qPCR technology, it can be used in the monitoring and development of early warning systems for HABs. From 2017 to 2020, samples were collected from 15 locations off the Chinese coast or from local sea areas. Based on the qPCR detection and analysis, the target species, *M. polykrikoides* (East Asian ribotype, EAr), was found in samples from Tianjin, Yangtze River estuary, and offshore Fujian (East China Sea). This is the first time that *M. polykrikoides* (EAr) was detected in the coastal waters of Tianjin. The results reveal a distributive pattern of *M. polykrikoides* (EAr) along Chinese coastal waters. It is helpful to predict the future diffusion trend of *M. polykrikoides* (EAr) in the China Sea and provides a practical case for the future construction of monitoring and warning systems for *M. polykrikoides* and HABs.

## 1. Introduction

*Margalefidinium polykrikoides* [1], formerly known as *Cochlodinium polykrikoides* [2], is an ichthyotoxic unarmored dinoflagellate that has caused mass mortalities of fish worldwide during blooms, with catastrophic impacts to aquaculture and local economies [1,3,4,5,6,7]. *M. polykrikoides* blooms are usually characterized by large spatial scale (10 to 100 km) and high-density aggregation (>10^6^ cells·L^−1^) [8]. These blooms are often accompanied by the production of strongly ichthyotoxic compounds, resulting in the death of a large number of marine organisms [9]. The ichthyotoxicity produced by *M. polykrikoides* can cause high mortality to marine organisms in a short period of time [9]. *M. polykrikoides* has been reported in tropical, subtropical, and temperate waters [10], such as the Gulf of California, United States of America [11], Korea [12], Japan [13], Middle East [5,14], Canada [7], and China [15]. Over the past three decades, harmful algal blooms (HABs) caused by *M. polykrikoides* have been spreading in Asian, Europe, and North American waters. In Korea alone, fishery losses associated with the blooms exceed $100M annually [16,17]. Compared with other countries, such as Japan and Korea, there are few reports regarding *M. polykrikoides* in Chinese coastal waters, and there is still a lack of reports on the detailed distribution of *M. polykrikoides* in the China Sea.

The rapid identification of the target species is imperative for timely monitoring and early warning of HABs [18,19,20]. Using conventional light microscopy (LM) and electron microscopy (EM) [21] to observe and identify microalgae is not only time-consuming and labor-intensive, but also requires high levels of taxonomic skill. Although these classical methods are still used today, it is difficult to achieve rapid detection and timely warning. In fact, for *M. polykrikoides* sharing similar morphological characteristics with other *Margalefidinium* species, it is difficult to distinguish and identify them from field samples [22,23,24]. Furthermore, after fixation with Lugol’s solution, glutaraldehyde, or other fixatives, some dinoflagellates may deform and even break. As unarmored dinoflagellate, the preservation time of *M. polykrikoides* is short in the fixative. This is not conducive to qualitative and quantitative analysis. However, researchers have never stopped exploring the development of rapid methods for microalgae detection. Using molecular methods to detect some HABs causative species can not only reduce the detection limit and improve the sensitivity and accuracy but it can also reduce the cost and processing time of each sample. Some molecular techniques, such as microarrays with molecular probes, restriction fragment length polymorphism (RFLP), high throughput sequencing, and fluorescent in situ hybridization (FISH-probes), have been developed for the detection and quantification of microalgae in the last three decades [25,26]. However, compared with the above detection methods, quantitative real-time PCR (qPCR) has higher sensitivity, specificity, and a more accurate quantitative effect [27,28].

In the past 20 years, qPCR has been used to detect and quantify microalgae. Park et al. resolved the intra-specific succession within *M. polykrikoides* populations in southern Korean coastal waters via the use of qPCR assays [10]. Park et al. developed a real-time PCR technique for detecting viable *M. polykrikoides* cysts in sediment [29]. Based on previous studies, Eckford-Soper and Daugbjerg developed a multiplex real-time qPCR assay that can simultaneously detect four marine toxic bloom-forming microalgal species [30]. Many scholars have conducted the qPCR quantitative analysis on major HABs causative dinoflagellates, such as *M. polykrikoides* [10], *Heterosigma akashiwo* [31], *Karenia mikimotoi* [32], *Prorocentrum donghaiense* [33], and *Alexandrium* [34]. Based on previous studies, effective primers have been developed and can be used to achieve the real-time quantitative amplification of target genes in S. Korea. In this study, qPCR was used to study the distribution of *M. polykrikoides* (East Asian ribotype, EAr) in the coastal areas of China.

## 2. Results

### 2.1. LM and Scanning Electron Microscopy (SEM)

**Figure 1:**
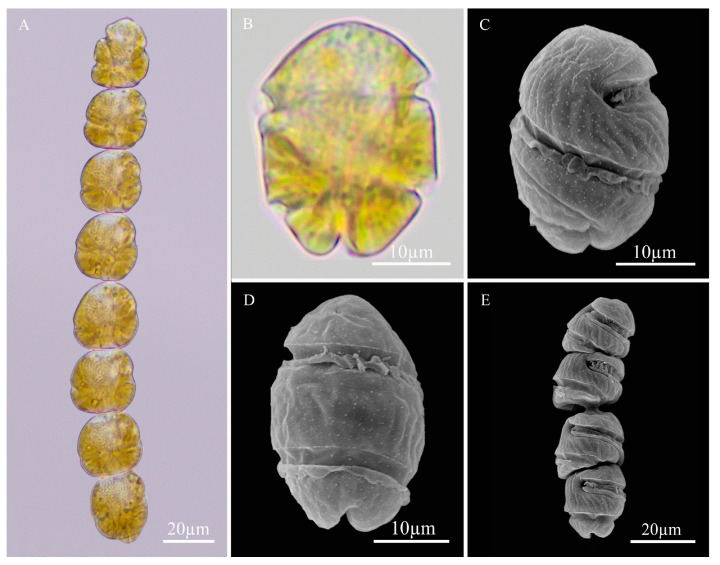
LM (**A**,**B**) and SEM (**C**–**E**) micrographs of *M. polykrikoides*.

*M. polykrikoides* (EAr), used to take LM micrographs, was obtained from the Department of Biotechnology at Sangmyung University (Seoul, Korea). *M. polykrikoides* used to take SEM micrographs was obtained from the East China Sea (ECS). The size of a single-cell of *M. polykrikoides* is 25–36 µm long and 16–25 µm wide (Figure 1). Under the LM, the cells form an eight-celled chain (Figure 1A). The single-cell of *M. polykrikoides* is conical at the tip and hemispherical at the bottom (Figure 1B–D).

### 2.2. Melting Curve and Standard Curve

**Figure 2:**
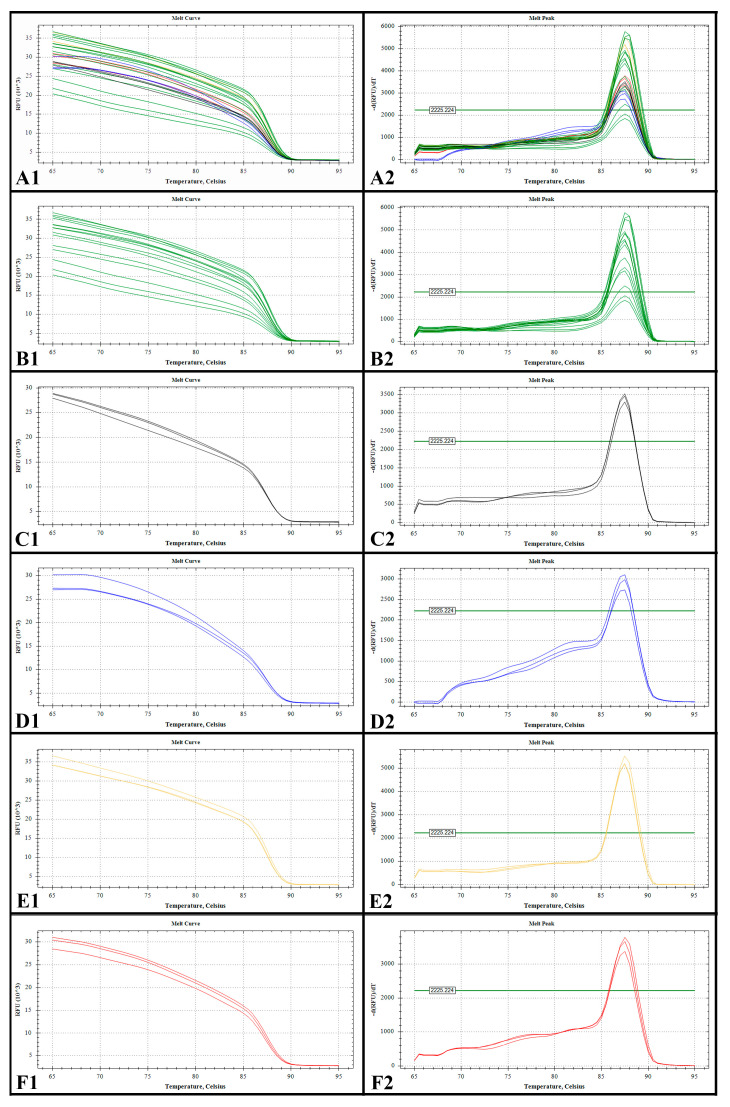
Melting curves obtained using the qPCR assay with DNA extracts from *M. polykrikoides* (EAr). (**A1**,**A2**): Standard sample and field samples. (**B1**,**B2**): Standard sample. (**C1**,**C2**): Field samples from Tianjin (S1). (**D1**,**D2**): Field samples from the Yangtze River estuary (S6, September). (**E1**,**E2**): Field samples from the Yangtze River estuary (S7, July). (**F1**,**F2**): Field samples from Fujian coastal (S11, May).

Each qPCR product had one informative melting curve. Analyzing the melting curve is useful for the detection of false positives due to primer-dimers or unexpected products. In this study, the melting temperatures were 88 °C and the melting curve showed narrow peaks, indicating that only the target sequence was successfully amplified (Figure 2).

The number of cells corresponding to serial 10 fold dilutions of the DNA extracts was 7.09 × 10^−3^, 7.09 × 10^−2^, 7.09 × 10^−1^, 7.09, and 70.9 cells·µL^−1^ (cell concentration dissolved in TEbuffer), respectively. There was a strong linear relationship between the C_T_ value and the log_10_ of the cells number. The regression equation was:

y = −3.2417x + 25.52 (R^2^ = 0.998) (1)

**Figure 3:**
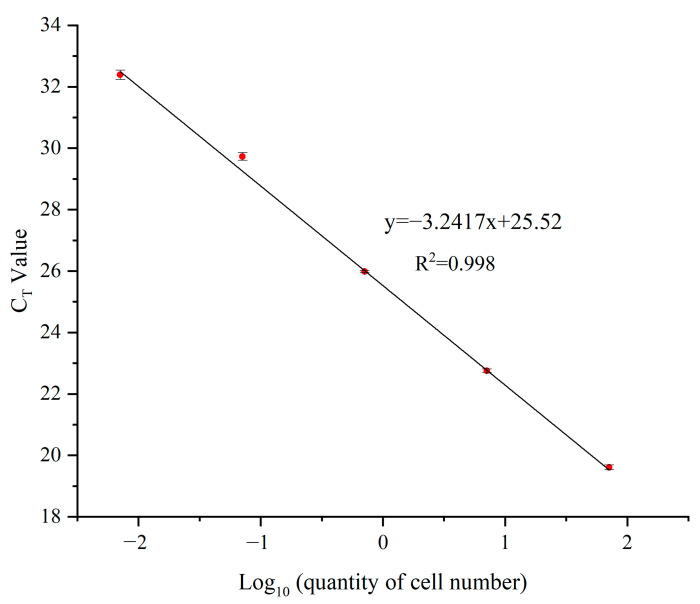
Standard curves of the qPCR assay using 10 fold dilutions of *M. polykrikoides* (EAr) DNA extracts.

The R^2^ values of all the standard curves were over 0.99 (Figure 3).

### 2.3. Application of qPCR to Field Samples

**Figure 4:**
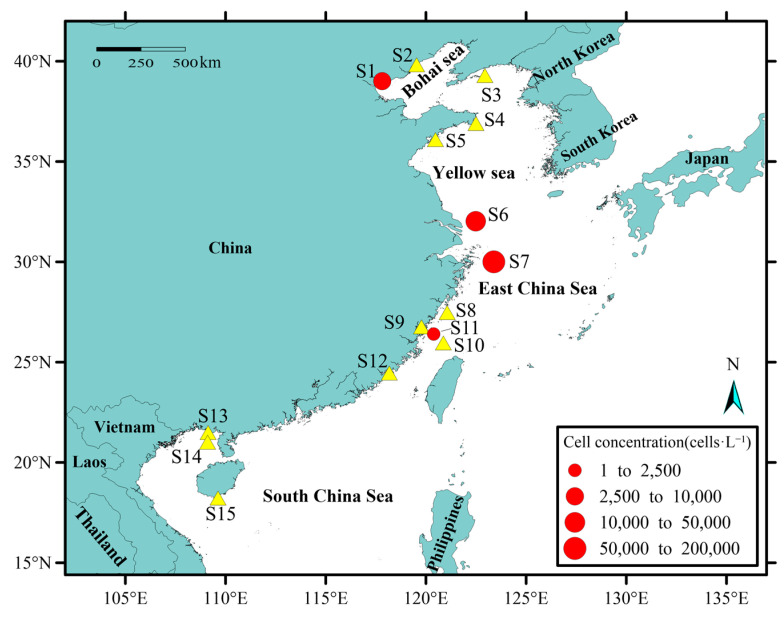
Distribution of *M. polykrikoides* (EAr) in coastal China based on qPCR assay. The Yellow triangle indicates the absence of *M. polykrikoides* (EAr), the red circles indicate the presence of *M. polykrikoides* (EAr), and the size of the circles indicates the concentration. Name of each station: (S1: Tianjin, S2: Beidaihe, S3: Zhangzi Island, S4: Rongcheng, S5: Qingdao, S6: Yangtze River estuary (September), S7: Yangtze River estuary (July), S8: Nanji Island, S9: Ningde, S10: Fujian coastal (April), S11: Fujian coastal (May), S12: Xiamen, S13: Beihai, S14: Weizhou Island, S15: Sanya.).

The applicability of the developed qPCR was tested on the field samples. Three repetitions were set for each sample. *M. polykrikoides* (EAr) was detected at four sampling sites, including Tianjin (S1), the Yangtze River estuary (S6, September), the Yangtze River estuary (S7, July), and Fujian coastal (S11, May) (Table 1). The site S11 showed the lowest abundance (1.5×10^3^ cells·L^−1^), while S7 displayed the highest concentration with 1.0 × 10^5^ cells·L^−1^. Overall, 3.6 × 10^3^ and 1.9 × 10^4^ cells·L^−1^ were detected at S1 and S6, respectively (Figure 4). The water temperatures at the stations where *M. polykrikoides* (EAr) was detected were 27.5 °C (S1), 25.4 °C (S6), 26.2 °C (S7), and 24.7 °C (S11), respectively. The average temperature was 25.9 ± 1.2 °C. The study results showed that *M. polykrikoides* (EAr) was detected in the Bohai Sea and the East China Sea, while no *M. polykrikoides* (EAr) was detected in the Yellow Sea and the South China Sea. The sampling time of the stations where *M. polykrikoides* (EAr) was detected was concentrated from May to September. Station S11, located in Fujian coastal, was sampled in May. Station S7, located in the Yangtze River estuary, was sampled in July. Station S1, located in Tianjin, was sampled in August. As time went on, *M. polykrikoides* (EAr) was detected from south to north.

### 2.4. Phylogenetic Tree

**Figure 5:**
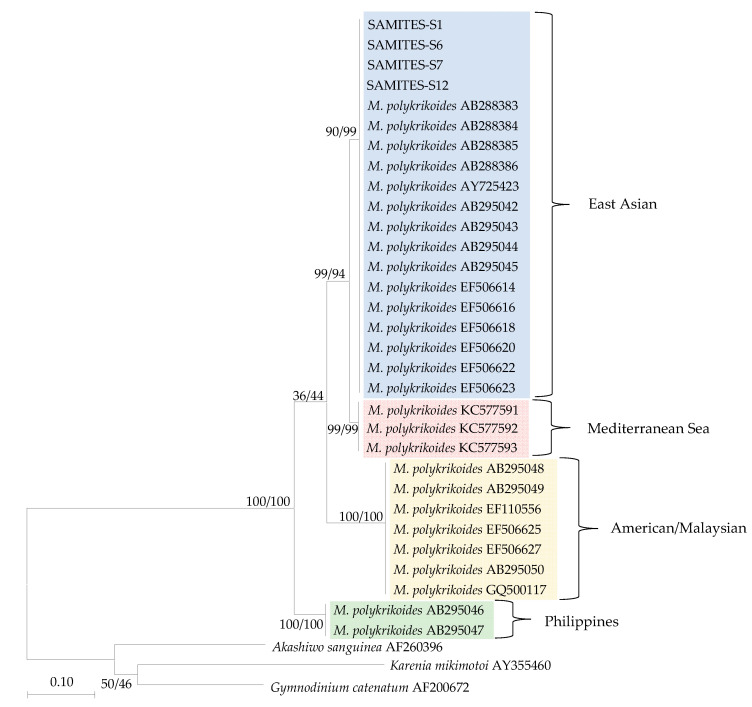
The percentage of trees in which the associated taxa clustered together is shown next to the branches. The tree is drawn to scale, with branch lengths in the same units as those of the evolutionary distances used to infer the phylogenetic tree. Bootstrap support (BS) values of the Maximum likelihood (ML) and neighbor-joining (NJ) analysis are given left and right, respectively. Accession numbers are indicated after species names. SAMITES: Succussed Amplified M. polykrikoides (EAr) In Tested Environment Sample.

The qPCR amplification products of field samples with positive results were sequenced, and all sequencing results were an identical sequence. In the clade of *M. polykrikoides*, specimens were clearly separated into four monophyletic sub-clades. One clade of *M. polykrikoides* (EAr) was composed of sequences collected from this study and other Asian seas, and all sequences in this clade were completely identical, which was well supported by bootstrap support (BS) values (neighbor-joining (NJ)/maximum likelihood (ML) = 90%/99%). The phylogenetic tree clearly shows the relationships of *M. polykrikoides* (EAr) and other ribotypes, including the Philippines ribotype, American/Malaysian ribotype, and the Mediterranean Sea ribotype (Figure 5).

## 3. Discussion

Although many molecular methods, such as microarrays with molecular probes [35], RFLP [36], high throughput sequencing [37], and FISH-probes [38], have been developed to detect microalgae, qPCR is more widely used to detect and quantify microalgae because of the high accuracy and good sensitivity [27,28,39]. Currently, qPCR has been applied in the field to detect and quantify harmful algae [40,41,42,43]. It may be difficult to detect and quantify microalgae by direct counting with LM at low densities of the target species, but based on the high sensitivity of qPCR, it is possible to detect and quantify the target species even at low densities (<10 cells·L^−1^) [10,44]. The copy numbers of the rRNA gene of dinoflagellates can be up to the order of 10000 [45]. Therefore, even if the extracted DNA contains less than one cell due to dilution, the rRNA gene can be amplified [10]. On the other hand, a high copy number can effectively eliminate PCR inhibitor interference in the qPCR process by diluting the extracted DNA. PCR inhibitors, such as mucopolysaccharides, phenolic compounds, humic acids, and heavy metals in field samples, may cause the qPCR results to be inaccurate or even false negative [46,47]. Based on Park et al. research foundation, the results of this study indicate that the qPCR primer (CPSF2-CPSR3) for the *M. polykrikoides* (EAr) has a strong specificity and is appropriate for the specific detection and quantification of *M. polykrikoides* (EAr) [10].

*M. polykrikoides* is present in tropical, subtropical and temperate waters [10]. Kim et al. [48] research results showed that *M. polykrikoides* examined in the laboratory exhibited its maximum specific growth rate of 0.41 day^−1^ at a combination of 25 °C and salinity of 34 psu, and optimum growth rates of >0.3 day^−1^ were observed at temperatures ranging from 21 to 26 °C and at salinities from 30 to 36 psu. It can be seen that most sea areas in China are suitable for the growth of *M. polykrikoides.* In 1993, Qi et al. reported the occurrence of red tide of *Margalefidinium* sp. in Quanzhou Bay, Fujian Province, which caused the death of a large number of marine organisms, but the cause species were not confirmed [15]. In 2009, *M. polykrikoides* was found in the Pearl River Estuary, South China Sea [49]. In 2014, morphological characterization and phylogenetic analysis of *M. polykrikoides* isolated from the ECS were carried out by Wang et al. [50]. In 2019, *M. polykrikoides* was detected in Jiaozhou Bay, Qingdao and demonstrated strong temporal preference with a sharp peak of abundance in early autumn (September), but failed to detect *M. polykrikoides* from January to May [51]. In this study, we also collected samples in coastal Qingdao on 10 May 2019. It is consistent with the research results of Liu et al. [51], we also failed to detect *M. polykrikoides*. This may be related to the low water temperature (16.9 °C). However, compared with countries such as Japan and Korea, there are still fewer reports on geographical distribution of *M. polykrikoides* in China’s coastal areas. In 1978, the first outbreak of red tide of *M. polykrikoides* occurred in the Yatsushiro Sea of Japan, and thereafter, the red tide caused by this species rapidly spread to the extensive waters along the coast of Japan and Korea [22,48,52]. Marine ecosystems are experiencing warming due to global climate change [53]. Seawater warming change the basal metabolic function and species distribution in microalgae [54]. In addition, ballast water is also an important reason for the spread of microalgae species [55]. In a time of global warming and increasingly advanced shipping, the rate of spread of harmful algal bloom species is increasing. Therefore, there is a possibility that *M. polykrikoides* could cause a massive outbreak in the China Sea. Timely warning is the key to face the outbreak of HABs. qPCR can achieve rapid detection and timely warning.

The results of this study show that *M. polykrikoides* (EAr) existed in the ECS and Tianjin coastal area, and their concentration can reach 1.0 × 10^5^ cells·L^−1^ at least in the ECS. In this study, field samples were collected from 15 locations, 3 of them were offshore sampling. *M. polykrikoides* (EAr) was detected in all three samples sampled offshore. However, *M. polykrikoides* (EAr) was detected in only 1 of the 12 samplings conducted near shore. One of the possible reasons why *M. polykrikoides* (EAr) were rarely found near shore may be due to the high turbidity of nearshore waters, which can affect the growth and distribution of *M. polykrikoides* (EAr). Blooms of *M. polykrikoides* are influenced by prevailing ocean currents [56]. Lee et al. research results showed that the outbreak of *M. polykrikoides* in coastal areas of Korea was influenced by the Tsushima Warm Current [52]. Large-scale transport of *M. polykrikoides* blooms by the Tsushima Warm Current also happened in the southwest Sea of Japan [57]. As a branch of the main stem of the Kuroshio in the northeastern waters of Taiwan Island, Taiwan Warm Current (TWC) is a high-temperature, high-salt current that exists year-round in the waters of Fujian and Zhejiang, China [58]. TWC carries high nutritive (phosphate) seawater to the Yangtze River estuary and plays an important role in the hydrology and climate of the ECS [59]. There is no report on the effect of TWC on *M. polykrikoides,* but there are many studies on the effect of TWC on *Prorocentrum donghaiense*. The research results of Dai et al. support the hypothesis that *P. donghaiense* blooms develop from the population at the TWC front in the ECS [60], suggesting the role of the ocean current front as a seed bank to dinoflagellate blooms. Zeng et al.’s research results showed that *P*. *donghaiense* blooms first occurred at the northern end of the Taiwan Strait and then moved northward and nearshore with the TWC [61]. Whether TWC will affect *M. polykrikoides* remains to be investigated. Although the effect of TWC on *M. polykrikoides* was not addressed in this study, it may be an important direction for future research on the distribution of *M. polykrikoides* in the coastal waters of China.

In this study, *M. polykrikoides* (EAr) was detected in the coastal waters of Tianjin for the first time. Since the first reported outbreak of *Cochlodinium* sp. in Quanzhou Bay in 1993 [15], this study confirmed the presence of *M. polykrikoides* (EAr) in the offshore of Fujian. *M. polykrikoides* has a maximum specific growth rate at 25 °C [48]. The water temperature at the time of the *M. polykrikoides* blooms mostly between 20 to 30 °C [62,63]. In this study, the water temperatures at the stations where *M. polykrikoides* (EAr) was detected were 27.5 °C (S1), 25.4 °C (S6), 26.2 °C (S7), and 24.7 °C (S11), respectively. The average temperature was 25.9 ± 1.2 °C. Some stations where *M. polykrikoides* (EAr) was not detected, such as S4, S5, S9, and S10, had water temperatures below 20 °C. Therefore, under suitable environmental conditions, those stations where *M. polykrikoides* (EAr) was not detected in this study may also exist *M. polykrikoides* (EAr). The sampling time of the stations where *M. polykrikoides* (EAr) was detected was mainlyfrom May to September. Following the time and season passing and changing, *M. polykrikoides* (EAr) was detected from south to north. It suggests that *M. polykrikoides* (EAr) is likely to keep moving northward as the water temperature rises. In March 2005, massive fish mortalities and water discoloration was reported off the western coast of Puerto Princesa, Palawan, Philippines; phytoplankton analysis revealed a near monospecific bloom of the dinoflagellate, *M. polykrikoides* [64]. There is a possibility that *M. polykrikoides* is expanding with seasonal changes from the low to latitude the high latitude sea area, though there still need for further study to clarify this issue.

## 4. Conclusions

In this study, qPCR was successfully applied to detect and quantify field samples along the Chinese coast. The target species, *M. polykrikoides* (EAr), was found in samples from Tianjin, the Yangtze River estuary, and offshore Fujian (East China Sea). This is the first time that *M. polykrikoides* (EAr) was detected in the coastal waters of Tianjin. Based on the accuracy, rapidity, and sensitivity of qPCR technology, it can be used in the monitoring and early warning system of HABs. It provides a practical case for the future construction of monitoring and warning systems for *M. polykrikoides* (EAr) and HABs.

## 5. Materials and Methods

### 5.1. Algal Cultures

The algal culture of *M. polykrikoides* (EAr) was obtained from the Department of Biotechnology at Sangmyung University (Seoul, Korea). The cultures were maintained in f/2 medium at 20 °C ± 1 °C under a light intensity of 65 µmol·Em^−2^·s^−1^ on a 12:12 h light-dark cycle. The f/2 medium was prepared as described in [65,66]. All seawater at a salinity of 31–33 psu was filtered through GF/F membranes (Whatman, Little Chalfont, UK) and then autoclaved at 121 °C for 30 min. Strains were subcultured with fresh f/2 medium at 20 day intervals to maintain healthy cultures.

### 5.2. LM and SEM

LM images of *M. polykrikoides* (EAr) in the exponential growth phase were recorded using an inverted microscope (BX53, Olympus, Tokyo, Japan) and analyzed using CellSens Standard 2.3 software (Olympus, Tokyo, Japan) [67]. SEM images were obtained in the following steps. *M. polykrikoides* obtained from ECS in the exponential growth phase was fixed with 2% glutaraldehyde at 4 °C for 4 h. Following the fixed samples were filtered and collected through a 3 μm polycarbonate membrane (Merck Millipore, Burlington, MA, USA), washed with distilled water, thoroughly removed all fixed reagents and sea salt, dehydrated with a graded ethanol series treatment (30, 50, 70, 90, 100, and 100%; 30 min per concentration), dried with a critical point drier (Joel Hi-Tech Co., Dalian, China), and gold-coated in a sputter coater. Lastly, the *M. polykrikoides* cells were observed and photographed under an SEM (TM-1000 Tabletop Microscope, Hitachi High-Technologies Co., Tokyo, Japan) [67,68].

### 5.3. Study Area and Field Sampling

**Figure 6:**
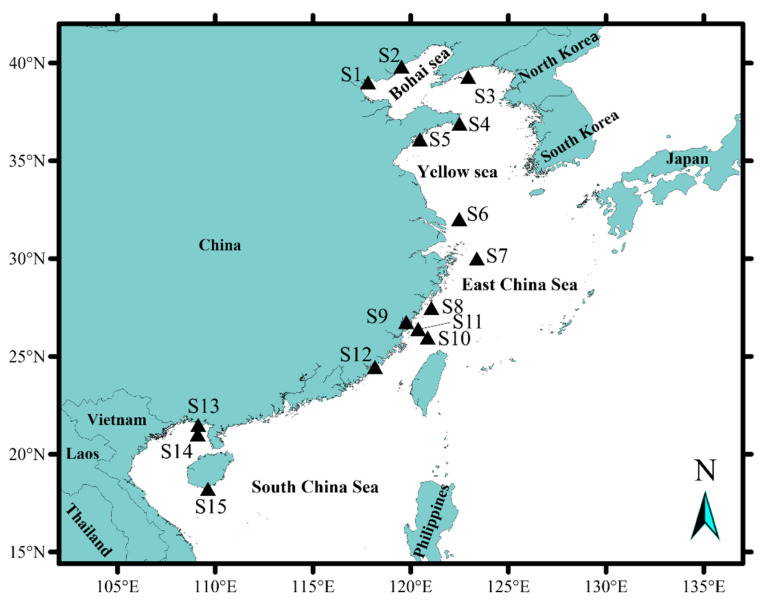
Map of sampling area. Name of each station: (S1: Tianjin, S2: Beidaihe, S3: Zhangzi Island, S4: Rongcheng, S5: Qingdao, S6: Yangtze River estuary (September), S7: Yangtze River estuary (July), S8: Nanji Island, S9: Ningde, S10: Fujian coastal (April), S11: Fujian coastal (May), S12: Xiamen, S13: Beihai, S14: Weizhou Island, S15: Sanya).

From April 2017 to November 2020, samples were collected from 15 locations of the Chinese coast or sea areas, including Tianjin, Beidaihe, Zhangzi Island, Rongcheng, Qingdao, Yangtze River estuary (July), Yangtze River estuary (September), Nanji islands, Fujian coastal (April), Fujian coastal (May), Ningde, Xiamen, Beihai, Weizhou island, and Sanya (Figure 6). The sampling time and location of each station are listed in Table 1. Seawater ranging from 100 mL to 1200 mL was filtered and collected via filtering with 0.2 μm Millipore filter membrane (Merck Millipore, Burlington, MA, USA). Seawater filtration volume (100–500 mL) were decided according to cell abundance and turbidity of seawater. The filters were placed in a 2 mL microtube containing 800 μL of 2% cetyltrimethylammonium bromide (CTAB) extraction buffer and then stored at −80 °C until DNA extraction.

### 5.4. DNA Extraction, PCR Amplification and DNA Sequencing

DNA extraction of samples was carried out with the CTAB method [69]. Based on the study of Park et al. [10], the East Asian ribotype of *M. polykrikoides* was confirmed by conventional PCR. The primers sequences for different ribotypes of *M. polykrikoides* were as follows: CPSF2: 5′-AACGCAAGTGTGAGTGTGAGTT, CPSR3: 5′-GGACCCACGATCAACCCA (EAr), PhiCPSF: 5′-TGCAAGTTTCAACCATCTCTCGC, PhiCPSR: 5′-GAAAGCAAGTTCAATCGACGGTTT (Philippines ribotype) and AMCPSF: 5′-CTCAATCGCCTTTCGCCTGAT, AMCPSR: 5′-ACCGGACACCTCGGATATGAT (American/Malaysian ribotype) [10]. The conventional PCR was carried out in a final volume of 20 µL containing 2 µL of 10× PCR buffer, 2 µL of dNTP (2.5 mM), 1 µL of each primer (0.1 mM), 12.8 µL of double-distilled water, 0.2 µL Takara Ex Taq polymerase (5U; TaKaRa, Osaka, Japan), and 1 µL of genomic DNA. Using T100^TM^ Thermal Cycler (Bio-Rad, Hercules, CA, USA), the PCR procedure was as follows: 95 °C for 5 min, 35 cycles at 95 °C for 30 s, 61.5 °C (EAr), 62 °C (Philippines ribotype), or 64 °C (American/Malaysian ribotype) for 30 s, 72 °C for 30 s followed by 72 °C for 10 min. PCR amplification products were analyzed by 2% agarose gel electrophoresis according to standard methods [70]. DNA sequencing was performed by Sangon Biotech (Shanghai, China).

### 5.5. qPCR and Standard Curve Construction

The species-specific qPCR primer used in this study for *M. polykrikoides* was developed by Park et al. [10]. DNA melting curve analysis was conducted using a CFX96 Real-Time PCR Detection System (Bio-Rad, Hercules, CA, USA). Quantitative Real-time PCR assays were performed in a total reaction volume of 20 µL, which contained 10 µL of 1 × SsoFast^TM^ EvaGreen^®^ Supermix (Bio-Rad, Hercules, CA, USA), 1 µL of each primer (0.1 mM), 1 µL of genomic DNA, and 7 µL double-distilled water. qPCR reactions were run using a CFX96 Real-Time PCR Detection System (Bio-Rad, Hercules, CA, USA) at 98 °C for 2 min, followed by 35 cycles at 98 °C for 5 s, then 55 °C for 20 s. The melting curve was increased from 65 °C to 95 °C in 0.5 °C increments, and each step was held for 5 s.

The specificity of a qPCR assay is determined by the primers and reaction conditions used. However, even with well-designed primers, it is always possible to produce primer-dimers or unexpected products. The specificity of the qPCR assay can be confirmed using melting curve analysis. The establishment of the standard curve was based on the linear relationship between the C_T_ value and the number of cells. Standard curves were constructed from DNA isolated from 150mL of culture harvested by filtration during the exponential growth phase. *M. polykrikoides* was counted using a Sedgewick Rafter counting chamber with an LM at 200 × magnification (CKX53, Olympus, Tokyo, Japan) and then filtered to collect the cells. The collected cells were extracted DNA according to the above method. DNA extracts were serially diluted 10-fold and used to construct standard curves.

### 5.6. Phylogenetic Tree

The sequences obtained by qPCR assay in this study and deposited in GenBank (https://www.ncbi.nlm.nih.gov/, accessed on 31 August 2021) were aligned with the sequences obtained from GenBank using BioEdit (North Carolina State University, Raleigh, NC, USA) (Version.7.0.5.3) [71]. The sequences obtained in this study were used together with those in Genbank to construct a phylogenetic tree and using *Akashiwo sanguinea, Karenia mikimotoi,* and *Gymnodinium catenatum* sequences as outgroup (Table 2) [72]. This analysis involved 34 nucleotide sequences. All positions containing gaps and missing data were eliminated (complete deletion option). There was a total of 146 positions in the final dataset. Evolutionary analyses were conducted in MEGA X (Pennsylvania State University, State College, PA, USA) (Version.10.2.4) [73]. The sequence obtained in this study is located in the D1-D2 region of the large subunit ribosomal RNA gene (LSU rDNA). ML and NJ phylogenetic tree based on partial LSU rDNA sequences showing the relationships of *M. polykrikoides* (EAr) and other ribotypes, including the Philippines ribotype, American/Malaysian ribotype, and the Mediterranean Sea ribotype.

### 5.7. Data Analysis

Amplification data were handled in a Bio-Rad CFX Maestro v 3.0 (Bio-Rad, Hercules, CA, USA), with Ct determination mode set to a single threshold and the baseline decided by baseline subtracted curve fit. Unknown cell concentrations were derived directly from the standard calibration curve by a Bio-Rad CFX Maestro v 3.0 (Bio-Rad, Hercules, CA, USA). Raw data were extracted to Microsoft Excel Professional Plus 2010 (Microsoft, Redmond, WA, USA) and OriginPro 2019b (Originlab Co., Northampton, MA, USA), where they were inspected manually.

## Figures and Tables

**Table 1:** Sampling site information (sampling date, latitude and longitude, and cell density of *M. polykrikoides* (EAr)).

Sea Area	Station	Location	Collection Date	Latitude (N)	Longitude (E)	Temperature (°C)	*M. polykrikoides* (EAr) (Cells·L^−1^)
Bohai Sea	S1	Dongjiang, Tianjin	29 August 2017	39.0070°	117.8204°	27.5	3.6 × 10^3^
	S2	Beidaihe, Hebei	30 August 2017	39.8241°	119.5336°	23.0	N/D
Yellow Sea	S3	Zhangzidao, Liaoning	17 September 2019	39.1188°	122.8238°	22.4	N/D
	S4	Rongcheng, Shandong	1 June 2019	37.9075°	122.4100°	17.5	N/D
	S5	Qingdao, Shandong	10 May 2019	36.0500°	120.3444°	16.9	N/D
East China Sea	S6	Yangtze River estuary	21 September 2020	32.0190°	122.4900°	25.4	1.9 × 10^4^
	S7	Yangtze River estuary	15 July 2020	30.0010°	123.3920°	26.2	1.0 × 10^5^
	S8	Nanji island, Zhejiang	28 July 2019	27.4650°	121.0605°	31.5	N/D
	S9	Ningde, Fujian	8 January 2019	26.7528°	119.7772°	15.3	N/D
	S10	Fujian coastal	29 April 2018	27.3037°	120.9138°	19.0	N/D
	S11	Fujian coastal	21 May 2018	26.4046°	120.3876°	24.7	1.5 × 10^3^
	S12	Xiamen, Fujian	12 April 2018	24.4563°	118.1713°	23.8	N/D
South China Sea	S13	Beihai, Guangxi	18 November 2018	21.4888°	109.1252°	25.7	N/D
	S14	Weizhou island, Guangxi	9 November 2020	21.0138°	109.0986°	22.4	N/D
	S15	Sanya, Hainan	9 October 2019	18.2233°	109.6222°	29.9	N/D

N/D: Not detected.

**Table 2:** List of strains examined in the present study and accession numbers for their LSU rDNA sequences.

Species	Ribotype	Accession No.	LSU Region	Strain	Location	Date	Isolator
*Margalefidinium polykrikoides Margalef*	EA	AB288383	D1–D6	IN1-ND104	Inokushi Bay, Japan	January 2005	H. Kawami
*M. polykrikoides*	EA	AB288384	D1–D6	OB7-ND3	Tachibana Bay, Japan	July 2002	M. Iwataki
*M. polykrikoides*	EA	AB288385	D1–D6	KG8-ND14	Kamigoto Is., Japan	August 2002	M. Iwataki
*M. polykrikoides*	EA	AB288386	D1–D6	USUKA	Usuka Bay, Japan	October 2003	T. Yamatogi
*M. polykrikoides*	EA	AY725423	D1–D3	-	Korea	-	-
*M. polykrikoides*	EA	AB295042	D1–D3	-	Off Mishima Is., Japan	August 2003	T. Baba
*M. polykrikoides*	EA	AB295043	D1–D3	IS8-ND70	Isahaya Bay, Japan	August 2003	M. Iwataki
*M. polykrikoides*	EA	AB295044	D1–D6	IN5-ND81	Inokushi Bay, Japan	May 2004	M. Iwataki
*M. polykrikoides*	EA	AB295045	D1–D6	KT8-ND109	Katagami Bay, Japan	August 2005	H. Kawami
*M. polykrikoides*	EA	EF506614	D1–D3	C.poly	Namhae, Korea	September 2000	C.K. Lee
*M. polykrikoides*	EA	EF506616	D1–D3	PP-3	Tongyong, Korea	September 2001	C.K. Lee
*M. polykrikoides*	EA	EF506618	D1–D3	PP-6	Busan, Korea	September 2001	C.K. Lee
*M. polykrikoides*	EA	EF506620	D1–D3	CP2002	Busan, Korea	August 2002	C.K. Lee
*M. polykrikoides*	EA	EF506622	D1–D3	CP2002-1	Namhae, Korea	August 2002	C.K. Lee
*M. polykrikoides*	EA	EF506623	D1–D3	HK	Hong Kong	-	-
*M. polykrikoides*	MS	KC577591	D1–D2	SC5	Tarragona Harbour, Catalonia	September 2012	A. Rene
*M. polykrikoides*	MS	KC577592	D1–D2	SC6	Tarragona Harbour, Catalonia	September 2012	A. Rene
*M. polykrikoides*	MS	KC577593	D1–D2	SC7	Tarragona Harbour, Catalonia	September 2012	A. Rene
*M. polykrikoides*	Ph	AB295046	D1–D3	MBCp	Manila Bay, Philippines	October 2004	J.R. Relox Jr.
*M. polykrikoides*	Ph	AB295047	D1–D6	OM7-ND59	Omura Bay, Japan	July 2003	M. Iwataki
*M. polykrikoides*	A/M	AB295048	D1–D8	cp1	Sabah, Malaysia	January 2004	A. Anton
*M. polykrikoides*	A/M	AB295049	D1–D7	cp2	Sabah, Malaysia	January 2004	A. Anton
*M. polykrikoides*	A/M	EF110556	D1–D3	CpFB-06-1	Long Island, NY, USA	August 2006	Y. Tang
*M. polykrikoides*	A/M	EF506625	D1–D3	CPCB10	Cotuit Bay, MA, USA	September 2001	D. Kulis
*M. polykrikoides*	A/M	EF506627	D1–D3	CPPV-1	Bahı’adeLaPaz, Mexico	-	L. Morquecho
*M. polykrikoides*	A/M	AB295050	D1–D8	PR107	Phosphorescence Bay, Puerto Rico	2005	C. Tomas
*M. polykrikoides*	A/M	GQ500117	D1–D2	CPDBC4	United Arab Emirates	-	-
*Akashiwo sanguinea*		AF260396	D1–D3	JL36	-	-	S. Morton
*Karenia mikimotoi*		AY355460	D1–D3	NOAA-2	Sarasota, FL, USA	-	-
*Gymnodinium catenatum*		AF200672	D1–D3	-	Spain	-	-

EA: East Asian; MS: Mediterranean Sea; Ph: Philippines; A/M: American/Malaysian.

## Data Availability

No new data were created or analyzed in this study. Data sharing is not applicable to this article.
